# A Tertiary Center Case Series on Postpartum Rectus Sheath Hematoma: An Unveiled Identity

**DOI:** 10.7759/cureus.58903

**Published:** 2024-04-24

**Authors:** Sakshi Agrawal, Yashaswi Pandey, Ishan Kumar, Lalit Kumar

**Affiliations:** 1 Department of Obstetrics and Gynecology, Institute of Medical Sciences, Banaras Hindu University, Varanasi, IND; 2 Department of Radiodiagnosis and Imaging, Institute of Medical Sciences, Banaras Hindu University, Varanasi, IND; 3 Department of Urology, Institute of Medical Sciences, Banaras Hindu University, Varanasi, IND

**Keywords:** pregnancy, maternal mortality, cesarean, postpartum, rectus sheath hematoma

## Abstract

Rectus sheath hematoma is a well-recognized, uncommon clinical entity and may not be the initial consideration when evaluating a postpartum patient with abdominal pain or mass. Here, we report three cases of postpartum rectus sheath hematomas (RSH) managed during the last three years. The mean age of the patient was 28 (25-30) years. All patients had a history of cesarean section and presented with pain and distension in the abdomen. The cesarean was performed eight days, one day, and three days in cases 1, 2, and 3, respectively, before presentation to the hospital. Two (Cases 1 and 3) of the three patients received conservative care and were discharged in stable condition. One patient (Case 2) who was operated on for RSH and hemoperitoneum expired due to multiorgan dysfunction syndrome (MODS). Our case series suggests that, depending on the severity of the hematoma and the hemodynamic condition of the patient, RSHs may require medical intervention ranging from conservative management to surgical treatment. Early diagnosis and intervention help prevent hazardous complications and prevent maternal morbidity and mortality.

## Introduction

Rectus sheath hematoma (RSH) is a rare medical condition causing pain in the abdomen that occurs when blood accumulates within the rectus sheath due to injury to inferior/superior epigastric vessels or tear of anterior abdominal wall muscles. It is the most prevalent primary non-neoplastic condition of the rectus muscle and sheath. They are typically found infra-umbilically in location and are frequently mistaken as abdominal tumors, inflammatory illnesses, or acute abdomens. It rarely happens on its own, even though the etiology includes pregnancy, abdominal surgeries open or minimally invasive techniques, port insertion, inadequate surgical hemostasis during wound closure, trauma, subcutaneous drug injections, anticoagulant therapy, hematological disorders, hypertension, coughing, and physical activity. It can also be a cause of abdominal pain after delivery [1]. Being relatively weaker, the posterior rectus sheath can result in indirect irritation of the peritoneum when a hematoma forms below the arcuate line. This irritation can manifest as abdominal pain and tenderness, mimicking the symptoms of an acute abdomen. It can also manifest as a lump in the abdomen, abdominal distension/peritonitis, bruising of the overlying skin, blood from the wound/drain site, fall in hemoglobin, and unconsciousness due to hemodynamic instability. Its diagnosis requires a high index of suspicion [1,2]. Spontaneous RSH has also been reported by Grigore M et al. and Eckhoff K et al. during pregnancy at 32 weeks and 26+3 weeks, respectively, which were managed conservatively and by surgical ligation of arterial bleeding vessels [3,4]. Berná JD had classified RSH into three grades depending on extent and severity. Type I RSH is intramuscular and does not cross the midline or dissect across fascial planes and results in slight to moderate abdominal pain. Type II may extend between the rectus muscle and transversalis fascia, can cross the midline, or can have bilateral involvement. Type III additionally can involve prevesical space and can cause hemoperitoneum. [5] Ultrasound (USG) has been reported to have a sensitivity of 85-96% and is useful in hemodynamically unstable patients, as well as pregnant patients, to maintain a strategic distance from ionizing radiation [6]. CT is superior to USG and is considered to be the gold standard, particularly in bigger hematomas where the relationship between the abdominal wall and the hematoma cannot be decided and diagnosis remains uncertain on USG [7].

RSHs, despite often resolving the nature of their own, can lead to severe complications in some cases. These complications may include compression of surrounding structures, compartment syndrome, infection, multiorgan dysfunction syndrome (MODS), hypovolemic shock, and in rare instances, death. The reported mortality rate for RSHs has been estimated at around 4%, although this figure can vary depending on factors such as the underlying cause, the patient's overall health, and the promptness of medical intervention. Conservative management with rest, analgesia, antibiotics, and cold compression is useful in milder cases. Large hematomas causing rupture/hemodynamic instability/peritonitis, expanding hematomas, and infected ones need surgical management. Evacuation followed by ligation of the bleeding vessel and drainage, if necessary, is usually enough to prevent further complications. Transcatheter embolization with thrombin, gelform, or coil, which is an alternative to surgery for conditions that do not respond to conservative management, may also be used [8].

So by keeping a high index of suspicion, implementing standardized postoperative monitoring protocols, and promptly investigating any concerning symptoms, healthcare providers can effectively detect and manage RSH promptly, minimizing the associated risks and optimizing patient care. We present a series of three cases that were diagnosed postoperatively as massive rectus sheath hematoma and their management. This case series will add to the literature available regarding the management of RSH.

## Case presentation

Case 1

A 30-year-old woman experienced lower abdominal pain for three days after undergoing a cesarean section eight days ago. The pain was severe in intensity, diffuse, and continuous, and was not shifting/referring/ or radiating to other sites. She also complained of a vague lump infra-umbilically. She received two units of packed red blood cell transfusion during the postoperative period and was discharged on the fifth day after surgery. There was no history of abdominal trauma or chronic cough. On physical examination, she was pale with a pulse rate of 106/min. Other vital signs were stable. During the abdominal examination, it was discovered that there was a low transverse edematous wound with intact sutures. The hypogastric and left iliac quadrant was occupied by a mass that was tender and felt like 10 x 7 cm, with some muscular stiffness present. In the right iliac fossa region, there was a tender mass measuring 6x5 cm that accompanied the bulky sub-involuted uterus.

**Figure 1:**
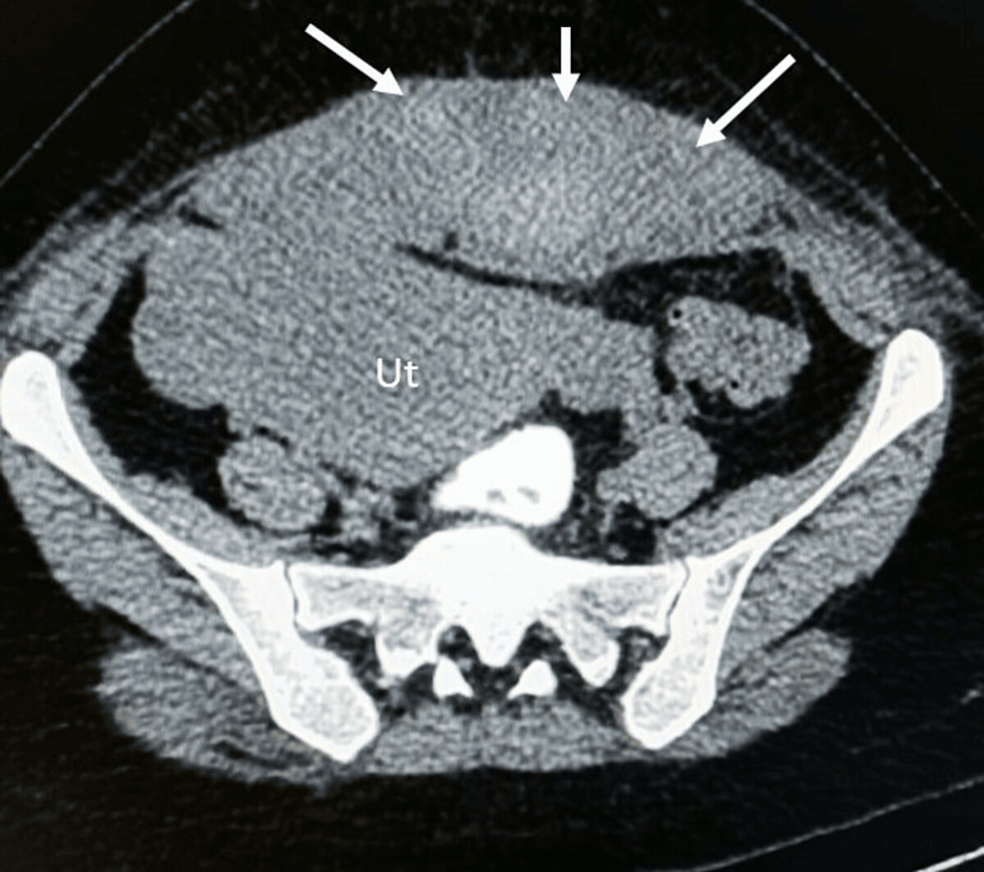
CT scan images of a 30-year-old female showing a large anterior wall hematoma (arrows) measuring 5.2 x 14 x 15.1 cm and extending bilaterally, splaying the abdominal wall muscles. The underlying bulky uterus is denoted by Ut.

Her hemogram, coagulation profile, liver function test, and renal function test were normal. Ultrasound was suggestive of a postpartum bulky uterus, and the bilateral rectus muscle appeared bulky and showed heterogenous hypoechoic content measuring 7.8 x 2.5 x 8.5 cm with a volume of 90.8 ml on the right rectus and measuring 11.5 x 4.3 x 11 cm with a volume of 287.6 ml in the left rectus, which were seen intercommunicating inferiorly with no obvious intra-peritoneal extension. On contrast-enhanced computed tomography (CECT) whole abdomen, a well-defined, hyperdense collection measuring 5.2 x 14 x 15.1 cm was noted in the rectus sheath extending from the level of the umbilicus up to pubic symphysis (Figure 1). The collection was breaching the linea alba and crossing the midline. There was no evidence of collection in the oblique muscle/intraperitoneal space. The patient was counseled about her morbidity and the pros and cons of conservative/surgical management. After informed consent, she was kept hospitalized for conservative management. She was recommended broad-spectrum antibiotics, analgesics, and anti-inflammatories. Serial ultrasound monitoring was done, which showed a resolving hematoma. After 15 days, complete resolution was attained and the patient was discharged.

Case 2

A 25-year-old unbooked female presented to the emergency department with acute-onset, severe abdominal pain, which was continuously associated with abdominal distension. The abdominal distension/lump in the abdomen was progressive and was involving the whole of the abdomen. The cesarean was performed outside one day back and the attendant stated that the drain was emptied twice with bloody fluid in it. On examination, her blood pressure was 90/60 mmHg, pulse rate 130 beats per minute, and respiratory rate 24 breaths per minute. Abdominal examination revealed tenderness, guarding and rigidity, bruising, and soakage present over the dressing, and on opening the dressing, frank bleeding was present from the stitch site, urine output was nil, and the patient was icteric and severely pale. All these features were suggestive of hemoperitoneum with peritonitis.

**Figure 2:**
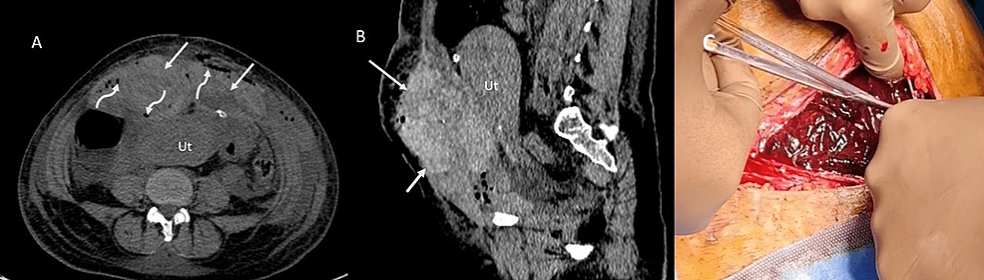
Non-contrast axial (A) and sagittal (B) CT images showing a large hyperdense and heterogeneous lesion (arrow) measuring 14x 4x 15.1 cm (approx. volume 1538 cc) involving bilateral rectus abdominis muscles suggestive of a large hematoma. The lesion is bulging posteriorly with peritoneal extension and mass effect over the uterus (Ut). Note the presence of multiple air foci within the lesion (curved arrows). Intraoperative pic showing large RSH in C. RSH: rectus sheath hematoma

Laboratory investigations revealed severe anemia (Hb: 4.6gm/dl), thrombocytopenia (platelet count: 74,500/μL), and elevated TLC (34,800/μL). A CT scan of the abdomen revealed a massive rectus muscle hematoma that measured 14 x 14 x 15 cm and had a volume of 1538.9 cc (Figure 2). Over time, the patient's coagulation profile, liver function test (LFT), and renal function tests (RFT) started to deviate, leading to a longer prothrombin time (PT) and activated partial thromboplastin time (aPTT). Fibrinogen levels were markedly reduced, and D-dimer levels were elevated. The coagulation profile indicated disseminated intravascular coagulation with multiple organ dysfunction syndrome (MODS).

For hemodynamic stabilization and close monitoring, the patient was immediately transferred to the intensive care unit. The initiation of coagulation support with fresh frozen plasma, cryoprecipitate, and platelet transfusions was initiated. Surgical intervention was considered. An RSH along with hemoperitoneum was identified intraoperatively, hemostatic sutures were taken, and hemostasis was secured. Over the next few days, the patient's condition deteriorated, with worsening organ dysfunction, including acute kidney injury, hepatic impairment, and respiratory compromise requiring mechanical ventilation. Despite aggressive treatment, the patient's condition continued to worsen, and she succumbed to MODS on the ninth day of hospital admission.

Case 3

**Figure 3:**
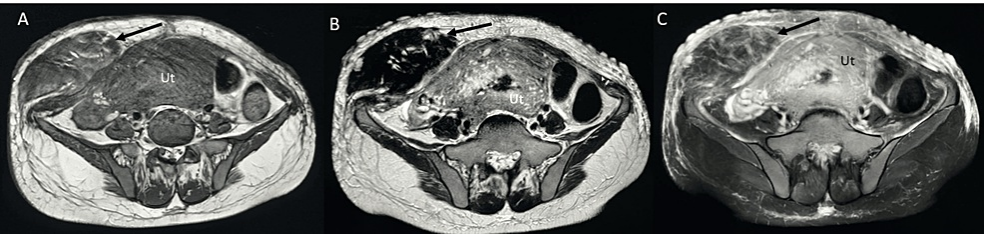
MRI T1W (A), T2 W (B), and fat-saturated T2W (c) images showing a large hematoma (arrows) involving the right side of the anterior abdominal wall involving subcutaneous tissue, right rectus abdominis, and right transversalis muscle. The lesion is isointense to the muscles on T1 and hypointense on T2-weighted images, suggesting acute hematoma. Note the bulky uterus (Ut).

An unbooked 29-year-old female P2L2 (Para 2 Live 2) was referred from the district hospital as a case of two previous cesarean sections with dull aching pain and swelling in the stitch line on postoperative day 3. On examination, her vital parameters were stable. She was operated on because of a previous cesarean with a contracted pelvis. She had no positive medical history of any illness. On admission, the patient was hemodynamically stable. Her blood pressure was 130/70 mmHg, her pulse rate was 96 beats per minute, and her respiratory rate was 18 beats per minute. On clinical examination, the patient had mild pallor. Per the abdomen examination, a 6x6 cm tender swelling was present on the right side of the stitch line with bruising of the overlying skin with interrupted suture over the stitch line. The uterus was 18 weeks in size. Her blood investigations were within normal limits. Ultrasound whole abdomen pelvis was suggestive of a postpartum bulky uterus and a heterogenous hypoechoic collection of volume 500 cc with internal septations without internal vascularity within the rectus sheath in the lower abdomen. Her CT whole abdomen pelvis was suggestive of a large hematoma involving the right side of the anterior abdominal wall (Figure 3). As the patient was hemodynamically stable, the decision for conservative management was taken after taking informed consent. Patient vitals were monitored. Blood parameters and ultrasound were repeated after one week and showed a resolving hematoma and improvement in hemoglobin. Clinically, her abdominal ache and lump progressively went away. The patient was discharged on postoperative day 10 in stable condition with proper follow-up advice.

Table 1 describes the history, detailed examination, and management of all three patients.

**Table 1:** Comparative analysis of all three cases of RSH RSH: rectus sheath hematoma

S.No.	Parameters	CASE 1	CASE 2	CASE 3
1	Age (years)	30	25	29
2	Booking status	Booked	Unbooked	Unbooked
3	Day of presentation	Day 8^th^	Day 2^nd^	Day 3^rd^
4	Symptoms	Pain	Pain and abdominal distension	Pain and swelling
5	Blood transfusion	Yes	No	No
6	Shock index	0.9	1.44	0.73
7	Hematoma	Bilateral	Hemoperitoneum	Unilateral
8	Type	II	III	I
9	Management	Conservative	Surgical	Conservative
10	Outcome	Improved	Mortality on Day 9	Improved
11	Discharge	Day 15	--	Day 10

## Discussion

The fibrous structure that makes up the rectus sheath is created by the tendinous extensions of the lateral abdominal muscles: the external abdominal oblique, internal abdominal oblique, and transversus abdominis, which encloses the rectus muscle. The first person to report an RSH in the United States was Richardson in 1857, although Hippocrates, Galen, and Leonardo da Vinci historically described rectus abdominis muscle hematoma [9].

In pregnant patients, RSH is most commonly attributed to non-traumatic factors such as increased intra-abdominal pressure from coughing, labor, or other activities. Blunt trauma, while it can cause RSHs, is relatively less common in pregnant patients compared to non-pregnant individuals [10]. It's crucial to acknowledge that although the inferior epigastric artery is a prevalent cause of bleeding in RSHs, other blood vessels within the rectus sheath can also contribute to hematoma formation. Additionally, factors such as anticoagulant medications, coagulopathies, and vascular abnormalities can increase the risk of RSHs by predisposing blood vessels to rupture or impairing normal blood clotting mechanisms [11,12].

RSH contributes to 1-2% of unexplained abdominal pain, with more prevalence among females. The female-to-male ratio is 2-3:1 [13]. Differentiating RSH from other causes of the acute abdomen can be challenging, particularly in the peripartum period when there are various physiological changes and potential complications associated with pregnancy and childbirth. Studies have reported maternal mortality rates ranging from 4% to 13% due to complications such as hypovolemic shock, organ dysfunction secondary to compression by the hematoma, or delays in diagnosis and treatment [8,14].

The reduction of both maternal and perinatal morbidity and mortality associated with RSH requires early recognition and intervention. The most frequent clinical presentation is a painful abdominal mass that can be felt. The other frequent symptoms include nausea and vomiting, ecchymoses on the anterior abdominal wall, signs of peritoneal irritation, fever, hypotension, and gradually increasing pallor. Clinical signs on examination like Cullen’s sign, Grey-Turner’s sign, Fothergill’s sign, and Carnet sign are to be looked for and once the clinical suspicion of rectus sheath hematoma is raised, the patient should be subjected to imaging modalities for the confirmation of diagnosis [15]. Ultrasound is used as the first step for establishing the diagnosis of rectus sheath hematoma. If the findings of ultrasound are inconclusive, the use of a CT scan for the location, extension, size of the hematoma, status of rectus abdominis muscle, and peri muscular tissue is suggested. Based on CT findings of severity of hemorrhage, rectus sheath hematoma is graded into three types [5,16].

MRI can be used to differentiate between anterior wall masses and chronic hematomas when CT findings are non-specific. The treatment plan for RSH is determined by the patient's hemodynamic stability, calculating the shock index, RSH size, presentation, and its extent and expansion [17]. Depending upon the severity of the hematoma and the factors above, the mode of treatment can be conservative or surgical. The decision of exploratory laparotomy depends on complications such as hematoma rupture with free peritoneal spillage/peritonitis, infection of RSH or myonecrosis, inability to resolve/rupture on conservative management, and hemodynamic instability [8,18]. The surgical process involves making an incision on the mass, removing the hematoma, washing it with local saline, identifying and ligating bleeding vessels if necessary, repairing the rectus sheath, and closure of the abdominal wall [19]. The conservative management strategy involves rest, painkillers, hemostatic compression, freezing packs, identifying predisposing conditions, and blood transfusions, if necessary.

## Conclusions

RSH is an uncommon cause of postpartum abdominal pain. Its non-specific and acute presentation can make it challenging to identify this missed identity promptly. Clinicians should be alert to the possibility of RSHs when patients present with acute pain and a lump in the abdomen, a fall in hemoglobin, and hemodynamic instability after cesarean, particularly when other common causes have been ruled out. So early history taking, a thorough physical examination, and the right imaging studies can contribute to preventing needless laparotomies. Incorporating RSHs into the differential diagnosis can result in timely diagnosis and management, ultimately improving patient outcomes. Most of the time conservative management suffices incorporating rest, painkillers, serial monitoring of the patient, intravenous fluid replacement, and blood transfusion if necessary.
